# TEP RNA: a new frontier for early diagnosis of NSCLC

**DOI:** 10.1007/s00432-024-05620-w

**Published:** 2024-02-19

**Authors:** Yuan Wang, Aiping Dong, Minhan Jin, Shirong Li, Yang Duan

**Affiliations:** 1 Clinical Laboratory, The First Affiliated Hospital of Weifang Medical University (Weifang People’s Hospital), Weifang Medical University, Weifang, 261000 Shandong China; 2 Department of Clinical Laboratory Science, Weifang Medical University, Weifang, 261000 Shandong China

**Keywords:** TEP RNA, Non-small cell lung carcinoma, Biomarker, Diagnosis

## Abstract

**Background:**

Non-small cell lung cancer (NSCLC) is the most common type of lung cancer (LC), which is the leading cause of tumor mortality. In recent years, compared with tissue biopsy, which is the diagnostic gold standard for tumor diagnosis, Liquid biopsy (LB) is considered to be a more minimally invasive, sensitive, and safer alternative or auxiliary diagnostic method. However, the current value of LB in early diagnosis of LC is not ideal, so it is particularly important to study the changes in blood composition during the process of tumorigenesis and find more sensitive biomarkers.

**Purpose:**

Platelets are a type of abundant blood cells that carry a large amount of RNA. In the LC regulatory network, activated platelets play an important role in the process of tumorigenesis, development, and metastasis. In order to identify predictive liquid biopsy biomarkers for the diagnosis of NSCLC, we summarized the development and function of platelets, the interaction between platelets and tumors, the value of TEP RNA in diagnosis, prognosis, and treatment of NSCLC, and the method for detecting TEP RNA of NSCLC in this article.

**Conclusion:**

The application of platelets in the diagnosis and treatment of NSCLC remains at a nascent stage. In addition to the drawbacks of low platelet count and complex experimental processes, the diagnostic accuracy of TEP RNA-seq for cancer in different populations still needs to be improved and validated. At present, a large number of studies have confirmed significant differences in the expression of TEP RNA in platelets between NSCLC patients and healthy individuals. Continuous exploration of the diagnostic value of TEP RNA in NSCLC is of utmost importance. The integration of NSCLC platelet-related markers with other NSCLC markers can improve current tumor diagnosis and prognostic evaluation systems, providing broad prospects in tumor screening, disease monitoring, and prognosis assessment.

## Introduction

### Background of non-small cell lung cancer

Lung cancer is the leading cause of cancer death worldwide, with non-small cell lung cancer (NSCLC) accounting for more than 80% of all lung cancers (Siegel et al. 2023). Although significant progress has been made in the treatment of lung cancer in recent years, the five-year survival rate of this disease is still not high, mainly due to the lack of effective early diagnostic methods. At present, the gold standard for tumor diagnosis is still tissue biopsy and pathologic diagnosis. However, it has been found that many patients refuse to undergo puncture surgery. Therefore, this method has significant limitations. Low-dose computed tomography (LDCT), as a screening diagnostic method for lung cancer, has been widely used in clinical practice; however, it has drawbacks such as high false-positive rates and radiation exposure (National Lung Screening Trial Research Team et al. 2011). Therefore, finding highly sensitive and specific tumor markers for early diagnosis, guiding treatment, and evaluating curative effects and prognosis is the key method to overcome malignant tumors and solve clinical diagnosis and treatment problems.

### Current status of liquid biopsy

**Fig. 1:**
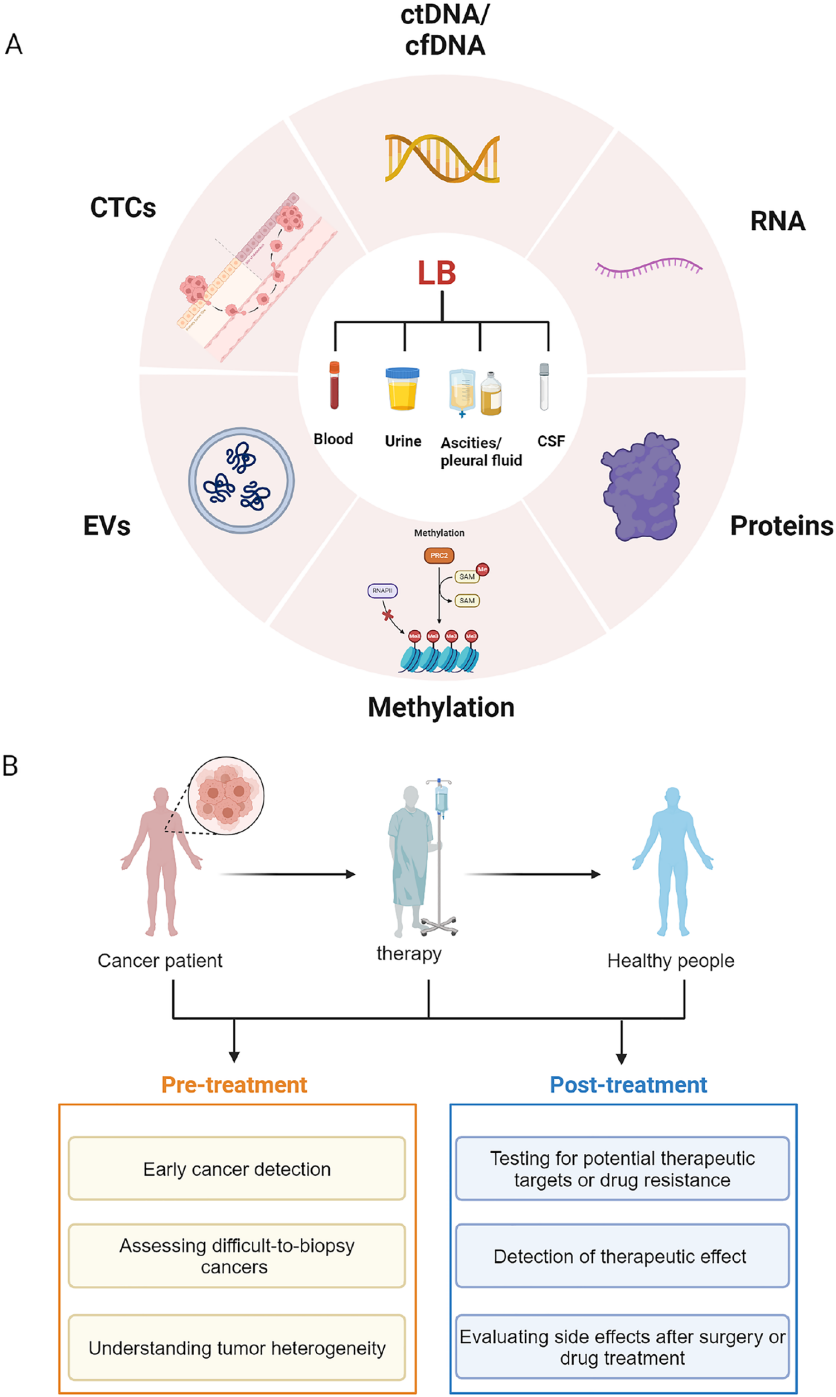
The classification of **a** sample types, test items of liquid biopsy and **b** clinical applications of liquid biopsy in tumor. Liquid biopsy (LB) is applied in clinical treatment and diagnosis by collecting patients’ blood, urine, pleural and ascitic fluids, as well as cerebrospinal fluid, and detecting their ctDNA/cfDNA, RNA, proteins, methylation products, EVs, and CTCs. LB can be applied to efficacy monitoring, disease prognosis, early cancer detection, monitoring of side effects after surgery or drug treatment, evaluating difficult to biopsy cancers, understanding tumor heterogeneity, and seeking treatment options. Created with BioRender.com

Liquid biopsy, as a new kind of diagnostic technique, has shown great potential in the field of tumor diagnosis in recent years (Cagle et al. 2013). It is minimally invasive, rapid, and highly sensitive and accepted by most patients. With the rapid development of LB technology, the detection of tumor markers has shifted from static detection to dynamic monitoring, and the research content and direction are gradually enriched. LB samples include blood, urine, chest and abdominal fluid, and cerebrospinal fluid, with blood being most commonly used in clinical practice. LB technology can be used not only to predict diseases, but also to detect efficacy, cancer types, evaluate side effects, understand tumor heterogeneity, and find the correct treatment methods (Fig. 1b). The detection objects of LB mainly include circulating tumor DNA (ctDNA)/circulating free DNA (cfDNA), circulating tumor cells (CTCs), extracellular vesicle (EV) released into the bloodstream from the primary tumor and metastatic sites, RNA, proteins and others (Fig. 1a) (Nikanjam et al. 2022). The expression of platelet-derived growth factor and transforming growth factor gene in lung cancer cells has been studied since 1988 (Söderdahl et al. 1988; Heldin et al. 1993). However, the current value of LB in early diagnosis of LC is not ideal, so it is particularly important to study the changes in blood composition during the process of tumorigenesis and find more sensitive biomarkers.

### Tumor-educated platelets (TEPs) as important biological sources for liquid biopsy

Platelets, as a type of abundant blood cells, play important roles in many physiological and pathological processes, including tumorigenesis. New therapeutic targets related to platelet function can be studied through platelet proteomics (Zufferey et al. 2012; Morris et al. 2022). Platelets are composed of fragments produced by megakaryocytes in the bone marrow. In the circulatory system, they function as highly reactive and secretory cell after produced by megakaryocytes for 5–7 days. In addition to its functions of hemostasis, thrombosis and innate immunity, platelets are also involved in the growth, extravasation, and metastasis of tumor cells, and have a significant impact on the treatment of tumor (Holinstat 2017; Haemmerle et al. 2018).

Furthermore, as observed in physical tests, the parameters of platelets can be affected by tumors. Platelet counts, which often tend to decrease during initial treatment and increase during relapse, reflect poor prognosis in solid tumors (Lin et al. 2014). For example, preoperative thrombocytosis is strongly associated with decreased survival rates in epithelial ovarian cancer (Hufnagel et al. 2020). By meta-analysis, Li et al. discovered a correlation between elevated platelet-to-lymphocyte ratio (PLR) and decreased overall survival (OS) or progression-free survival (PFS) among patients diagnosed with advanced cancer (Li et al. 2018). PLR can be used as a prognostic predictor of non-small cell lung cancer according to the study conducted by Liu et al. (Liu et al. 2019a). Recently, Li et al. undertook a retrospective analysis to investigate mean platelet volume (MPV), platelet count, survival rate, and risk of immune-related adverse events (irAEs) in patients treated with immune checkpoint inhibitors (ICIs). The results indicate a significant correlation between the alteration in MPV following one cycle of pembrolizumab treatment and both the overall survival and occurrence of irAEs in NSCLC patients (Li et al. 2023). MPV can also serve as a convenient prognostic diagnostic marker for locally advanced non-small cell lung cancer (Pyo et al. 2016; Sakin et al. 2019). Some scholars believe that there is an increase in MPV among patients with early-stage lung cancer, whereas it decreases in patients with advanced lung cancer (Sabrkhany et al. 2017; Goksel et al. 2021). Nevertheless, several scholars have discovered that the MPV among individuals diagnosed with lung cancer is comparatively lower than that observed in individuals who are unaffected by the disease (Şahin and Aslan 2018). The study discrepancy may be attributed to variances in cancer type and patient numbers across different stages. However, these parameters are influenced by additional inflammations. There is a requirement for an exploration aimed at identifying diagnostic markers that exhibit a higher degree of specificity.

### The biogenesis and function of the TEP RNA

Platelets are non-nucleated and abundant cellular debris derived from megakaryocytes (MK) of bone marrow stem cells, participating in the process of hemostasis, promoting angiogenesis, and inducing inflammation and immune response. The production of platelets is driven by the interaction of several transcription factors (Grozovsky et al. 2015). When the number of platelets in the blood is low, megakaryocytes are activated. Thrombopoietin (TPO) produced by the liver induces platelets formation by stimulating the thrombopoietin receptor (TpoR/MPL) in megakaryocytes. In reactive thrombopoiesis, IL-6 promotes proplatelets formation by increasing TPO level (Kaser et al. 2001). Even in the absence of nuclear structures, platelets contain various RNAs which undergo changes when MK become proplatelet cells. In this process, RNA-binding protein (RBP) plays important roles (Singh 2021). The RNA-binding protein SRSF3 distributes MK RNA into platelets and mediates RNA processing for MK gene regulation and platelet biogenesis (Heazlewood et al. 2022). Platelets have no nuclei, no DNA, and are rich in coding RNA (mRNA) and non-coding RNA (ncRNA). Among them, mRNA, miRNA, circulating RNA, and long-strand RNA are often used in tumor diagnosis and treatment. Studies have shown that RNA can be transferred to hepatocytes, vascular endothelial cells, macrophages, and tumor cells through platelets, and the integrated RNA can be translated into protein (Xia et al. 2018). TEP miRNA regulates gene expression, leading to changes in receptor cell function (Tran et al. 2020). In addition, some studies have confirmed that changes in platelet miRNA expression can affect platelet aggregation, antibacterial ability, and endothelial cell function (Qu et al. 2020). In the tumor microenvironment, platelets act as highly dynamic cells that communicate with tumor-related RNA transfer and influence the environment (D’Ambrosi et al. 2021a).

In this article, we will introduce the application of TEPs with tumor high driving phenotype in the diagnosis of NSCLC. It mainly includes the following aspects: (1) the historical interactions between platelets and tumors; (2) the clinical applications of TEP RNA in tumors; (3) the diagnosis, prognosis and treatment of NSCLC and TEP RNA; and (4) the method for detecting TEP RNA of NSCLC.

## The historical interplay between platelets and cancer

### The role of TEPs in tumors

**Fig. 2:**
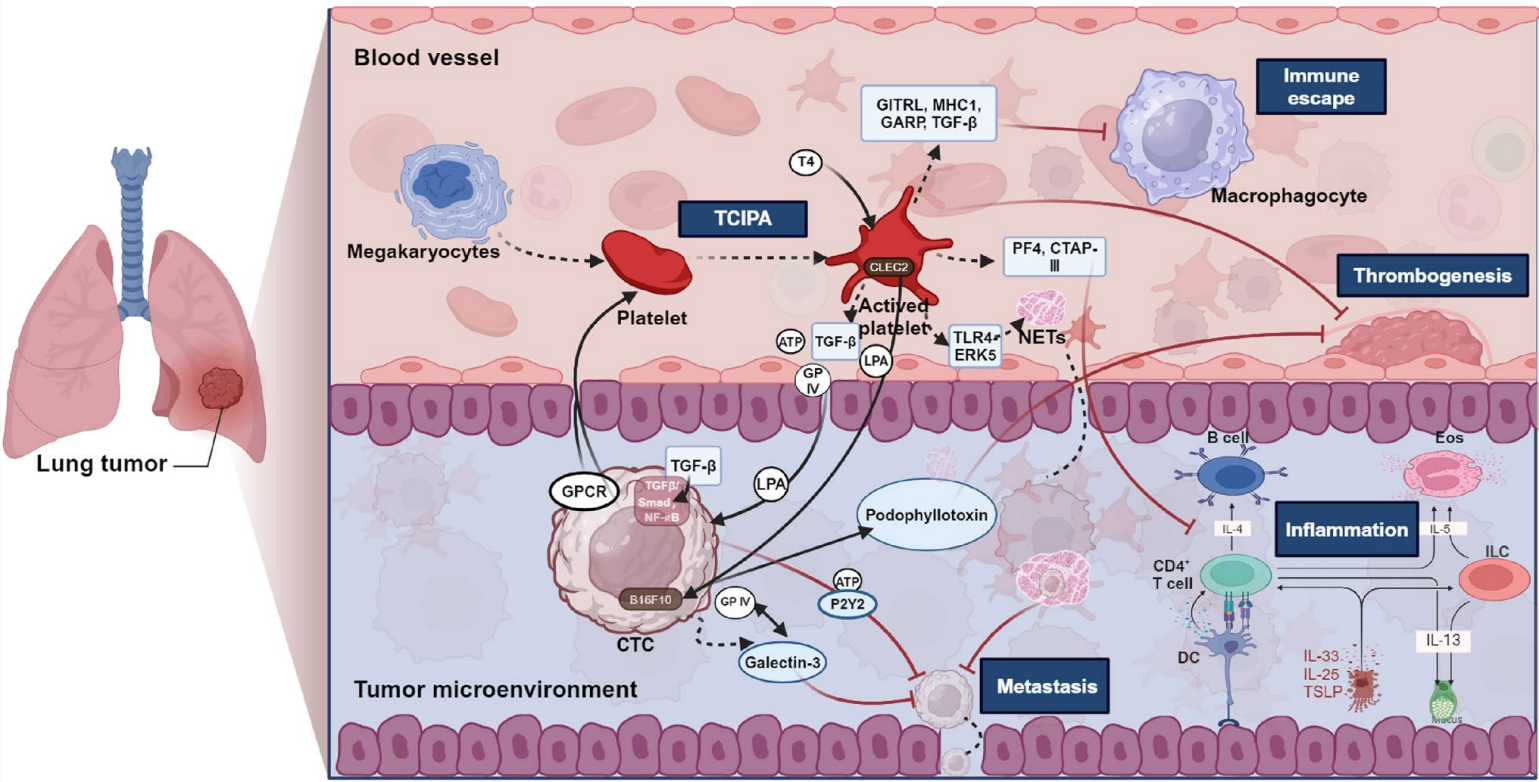
Interactions and mechanisms between platelets and tumor cells in tumor patients. Platelets are composed of fragments of megakaryocytes, and can be activated and undergo TCIPA under the action of GPCR and CTC. On the one hand, ATP, TGF-β, LPA, GP IV, and NETs released by activated platelets can act on the tumor microenvironment and affect tumor metastasis. On the other hand, activated platelets release GITRL, MHC1, GARP, TGF-β, PF4, and CTAP-III which participate in the occurrence of tumor-related inflammation and immune escape of tumors. The high expression of CLEC2 in activated platelets affects the synthesis of podophyllotoxin and thrombosis. Created with BioRender.com

TEPs play direct and indirect roles in the occurrence, development, and metastasis of tumors (Fig. 2). Firstly, platelets are activated and directly interact upon encounter with circulating tumor cells (CTC). Then activated platelets aggregate around tumor cells, forming platelet–tumor cell complexes to evade immune system clearance. After determining the relationship between platelets and cancer cells by Dyngo4a treatment and flow cytometry detection, the researchers found that cancer cells could uptake platelets by phagocytosis and recycling platelet membrane proteins (Martins Castanheira et al. 2022).

Second, activated platelets also release various factors, indirectly promoting tumor growth, angiogenesis, metastasis, and immune escape (Mezouar et al. 2014; Schlesinger 2018; Liu et al. 2021). Platelet factor 4 (PF-4/CXCL4) and connective tissue-activating peptide III (CTAP-III) are two platelet-related chemokines which regulate tumor angiogenesis and inflammation in the tumor microenvironment, thereby regulating tumor growth (Pilatova et al. 2013). Research has found that CLEC-2 is almost specifically expressed in human platelets/megakaryocytes. In B16F10 cells, CLEC-2 regulates the expression of podophyllotoxin and interacts with it, thereby regulating the formation of cancer-related thrombosis (Suzuki-Inoue 2019). Platelet TLR4 and ERK5 expression is significantly increased after surgical stress, and the platelet TLR4-ERK5 axis captures CTCs by releasing neutrophil extracellular traps (NETs) and promotes the distant metastasis of CTCs (Ren et al. 2021). Thyroid hormone T4, as a pro-metastasis factor, is a major ligand for the hormone receptor on tumor cells and platelet integrin αvβ3. As T4 activates integrins to promote platelet aggregation and degranulation, platelet ATP is released and promotes cell proliferation, invasion and metastasis (Davis et al. 2020). Furthermore, Platelet-derived ATP can also promote cancer cell metastasis through the P2Y2 receptor (Schumacher et al. 2013). Researchers found that platelet-derived TGF-β contacts with platelet-tumor cells and co-activates the TGF-β/Smad and NF-κB pathways in CTCs, leading to the transition to an invasive mesenchymal-like phenotype and enhancing the metastasis of cancer cells in vivo (Labelle et al. 2011). Platelet glycoprotein VI can also promote metastasis through interacting with cancer cell-derived galectin-3 (Mammadova-Bach et al. 2020). Therefore, it can be seen that we may explore the development of cancer cells by studying the proteome, transcriptome, and secretome of platelets ingested by cancer cells.

Finally, factors released by platelets not only promote the survival and migration of tumor cells in the bloodstream, but also provide a protective barrier for tumor cells to prevent immune surveillance. By generating GITRL, MHC1, GARP and TGF-β, TEPs can reduce the immunogenicity, immunoreactivity, and toxicity of NK cells, hence avoiding immune surveillance (Kataki et al. 2002; Placke et al. 2012; Li et al. 2022a).

The application of platelets in tumors is receiving increasing attention. Researchers have found that tumor-associated platelets (TAP) which block nanoparticles can alleviate cancer metastasis (Li et al. 2022b). Biomimetic nano-NOS mediated local NO can inhibit TEPs activation, destroy tumor’s vascular barrier, and exert anticancer effect (Ma et al. 2020). The TLR agonist, resiquimod (R848), enhances anti-tumor immunity in solid tumors through local delivery of platelet membrane-coated nanoparticles (PNP-R848) (Bahmani et al. 2021). TEPs has been proposed as a potential target for reducing the incidence rate, metastasis, and mortality of cancer (Mezouar et al. 2016).

### The role of CTCs on TEPs

Platelets are one of the most abundant cells in the blood circulation, second only to red blood cells, and are known as “circulatory sentinels”. They absorb and store a large number of proteins, cytokines, and specific receptors during circulation (Tran et al. 2020). These “platelet reservoirs” affect platelet count, function, RNA, and proteome changes.

During the development and metastasis of cancer, platelets are activated and functioning. First of all, tumor cells can alter the characteristics of platelets in turn. For example, the predictive significance of platelet counts and platelet lymphocyte ratio (PLR) in cancer diagnosis has been fully confirmed (Li et al. 2018). In addition, with changes in the tumor microenvironment, cancer also affects platelet RNA and protein profiles through changes in MK (Stone et al. 2012). In the study of tumor and platelet proteome, we find that six proteins (RNF100, CTSG, PGLYRP8, RPL100, S9A1, S1A432) are up-regulated, and two proteins (GPX1, TNS1) are downregulated in cancer patients (Walraven et al. 2021). Numerous DEG and bioinformatics analyses have confirmed that platelet RNA expression profiles play an important role in lung cancer metastasis and can serve as a mechanism for further research on tumor metastasis.

Driven by cancer-related proteins and cytokines, highly sensitive platelets act as the "first responders" of cancer metastasis, constantly responding to the occurrence and development of tumors. Tumor host angiogenic regulatory proteins can be selectively absorbed by platelets and participate in the regulation of platelet angiogenesis (Klement et al. 2009). Immunosuppressive cells, especially tumor-associated macrophages (TAMs), exist in the tumor microenvironment (TME). The consumption of TAMs enhances local and systemic platelet-mediated anti-PD-1 delivery effects by affecting platelet activation and aggregation, thereby inhibiting post-operative tumor recurrence. In addition to numerical changes, tumors also induce platelet dysfunction, thereby promoting thrombosis, which is known as tumor cell-induced platelet aggregation (TCIPA). During the TCIPA process, platelets are "educated" by tumor cells, further promoting tumor progression. Researchers showed that that adhesion to tumor cells expressing GPCR (CD97) receptors can promote the activation of platelet and the release of lysophosphatidic acid (LPA). LPA increases the invasive potential and vascular permeability of tumor cells, promoting tumor development through endothelial cells migration (Ward et al. 2018). By constructing a mouse model of in situ cancer (injection of 4T1 cells) and conducting in vitro cell experiments, Kassassir et al. found that platelet activation and platelet cancer cell aggregation rate increased with tumor progression; in addition, platelet activation and reactivity are closely related to lung metastasis and extrahepatic hematopoiesis (Kassassir et al. 2020).

## Clinical application of TEPs

### Application of platelet proteins in tumors

Platelet proteins originate from various sources including proteins present in debris from megakaryocytes, proteins internalized in the extracellular environment, and proteins that undergo translation and synthesis within the platelet ribose. Due to the fact that tumor cells possess the ability to stimulate platelet activation and release a significant quantity of regulatory proteins, numerous scholars have undertaken research on the simultaneous investigation of platelet proteins for the purpose of tumor diagnosis and treatment.

The concurrent detection of vascular endothelial growth factor (VEGF), platelet-derived growth factor (PDGF), and PF4 in platelets has the potential to serve as a diagnostic tool for the detection of colorectal cancer (Peterson et al. 2012). In addition, the application of platelet protein expression profiling can also serve to differentiate benign adnexal lesions from the diagnoses of ovarian and lung cancer, as well as aid in prognosis monitoring and diagnosing pancreatic cancer (Lomnytska et al. 2018; Sabrkhany et al. 2018). These studies have established a strong groundwork for the clinical application of platelet-derived proteins in the context of tumors.

Moreover, the utilization of platelet proteins extends beyond just the detection of tumors; it also encompasses their therapeutic capabilities. The investigation of platelet proteins’ impact on tumor progression has emerged as a significant topic in the field of TEPs research. Kuznetsov et al. discovered that platelets possess the ability to absorb proteins secreted by tumors and subsequently disperse them among other “potential tumors” in order to facilitate the advancement of tumor development and metastasis (Kuznetsov et al. 2012). In addition, Kerr et al. discovered that bone metastatic proteins derived from tumors, such as transforming growth factor b-1 and matrix metalloproteinase-1, were absorbed by platelets and subsequently released into the bone microenvironment via platelet α particles (Kerr et al. 2013). In the future, tumors may be effectively treated or even thwarted by the exertion of platelet proteins.

### Clinical monitoring of tumors by TEP RNA

Platelet RNA can additionally serve as a means for clinical monitoring during tumor therapy. Since the protection of tumor-derived transcripts by platelets from degradation by circulating plasma-derived ribonucleases and their continuous accumulation in TEPs, TEP RNA can be used to monitor dynamic changes in tumor activity. On the one hand, it can be used to monitor the progress of tumor treatment. On the other hand, it can also contribute to the exploration of acquired mutation sites that result in chemoresistance. In the diagnosis and treatment of glioblastoma, TEP RNA serves not only as a tool for monitoring the progression of glioblastoma, but also for distinguishing between false-positive progression and true progression (Sol et al. 2020). Since the effective crizotinib treatment for EML4-ALK has been observed to decrease the levels of EML4-ALK transcripts in TEPs during the treatment of NSCLC patients, it becomes possible to monitor TEP RNA to comprehend the drug therapy in these individuals with NSCLC (Nilsson et al. 2015). Park et al. has exhibited that the successive collection of TEP RNA adds predictive value in detecting EML4-ALK mutations, thereby enabling the prediction of favorable and unfavorable outcomes for ALK1 NSCLC patients who are undergoing treatment with alectinib or crizotinib (Park et al. 2019). Despite the fact that the current detection level of gene mutation of chemotherapy resistance by TEP RNA method is not as proficient as the conventional approach (Park et al. 2019; Brinkman et al. 2020), the utilization of the novel digital PCR technology can enhance both sensitivity and specificity in this domain of detection (Olmedillas-López et al. 2017).

### Current studies of TEP RNA in tumors (excluding NSCLC)

Compared with traditional tumor markers and circulating tumor RNA, TEP RNA is tumor-specific RNA with higher accuracy and specificity. There is a wealth of research on the applications of TEP RNA in tumors other than NSCLC (Table 1). By sequencing and analyzing 283 platelet samples, Best et al. concluded that TEP RNA can accurately distinguish cancer patients from normal individuals (96% accuracy) and locate six different types of primary tumors (71% accuracy) (Best et al. 2015). Specifically, TEP-miR-4435-1 is highly expressed in HBC, glioblastoma and PAAD, and low expressed in breast cancer, so it can be used for screening different cancers. In 2020, he continued to analyze the diagnosis of TEP RNA in pan cancer and found that one can use the information provided by TEP splicing RNA patterns and cfDNA patterns of mutations or methylation in plasma to locate pan cancer (Wurdinger et al. 2020). Veld et al. established a platelet RNA library, developed and tested Seq algorithms for 18 pan cancer cells, and concluded that platelet RNA can be used for blood-based cancer detection, staging, and identification of tumor sites of origin (Veld et al. 2022). In cluster analysis and principal component analysis (PCA), it was found that MT-RNR2, MT-RNR1, TMSB4X, B2M, and MTND1 can distinguish ovarian cancer from healthy individuals (Gao et al. 2023). Sarcoma, as a rare heterogeneous tumor disease, can also be detected through highly sensitive and specific TEPs PSO enhanced thrombus sequence analysis (Heinhuis et al. 2020). SNORA58, SNORA68, and SNORD93 were significantly upregulated in TEPs of ESCA patients (Zhang et al. 2023). Recently, Miao et al. found that TEP miRNA affects platelet activity, PMPs production, thrombosis risk, immune cell function, and sensitivity to chemotherapy drugs and aspirin by regulating platelet activity and megakaryocyte maturation and differentiation (Miao et al. 2021). In addition, platelets are considered to be potential targets for cancer therapy. The platelet CLEC2–Podoplanin axis can be used as a target in the treatment of oral cancer (Hwang et al. 2021). Myeloproliferative neoplasm (MPN) is a rare malignant hematological disease with great difficulty in diagnosis. Girardot et al. took the first step in the progress of TEP RNA and MPN diagnosis. He found that miR-28 was a negative regulator of myeloid cytokine receptors, targeting not only the translation of MPL and E2F6 proteins, but also their terminal differentiation in CD34 derived megakaryocytes (Girardot et al. 2010). Notably, a few years later, he reported that TEP miR-28 was highly expressed in a subgroup of MPN patients, which may be related to the constitutive activation of STAT5 (Girardot et al. 2015). In ovarian cancer, PMPs play a role through sPLA2-IIa mediated miR-939 detachment, leading to epithelial–mesenchymal transition and tumor progression (Tang et al. 2017). Wang et al. found that TEP miR-34c-3p and TEP miR-18a-5p were highly expressed in nasopharyngeal carcinoma patients through real-time fluorescence quantitative PCR detection and ROC analysis (Wang et al. 2019).

**Table 1:** Roles of TEP RNA in the occurrence and progression of other tumors

Tumor type	TEP RNA	Dysregulation type	Function	References
HBC	miR-4435–1	Up	–	Best et al. (2015)
GBM	miR-4435–1	Up	–	Best et al. (2015)
PAAD	miR-4435–1	Up	–	Best et al. (2015)
BC	miR-4435–1	Down	–	Best et al. (2015)
OC	MT-RNR2	Up	–	Gao et al. (2023)
	MT-RNR1	Up	–	Gao et al. (2023)
	TMSB4X	Up	–	Gao et al. (2023)
	B2M	Up	–	Gao et al. (2023)
	MTND1	Up	–	Gao et al. (2023)
	CLEC2	–	CLEC2-PDPN axis mediates the formation of TCIPA	Hwang et al. (2021)
	miR-939	–	Induces EMT and enhances cancer progression	Tang et al. (2017)
ESCA	SNORA58	Up	–	Zhang et al. (2023)
	SNORA68	Up	–	Zhang et al. (2023)
	SNORD93	Up	–	Zhang et al. (2023)
MPN	miR-28	Up	Plays a negative role on proplatelet formation	Girardot et al. (2010)
NPC	miR-34c-3p	Up	Has significant clinical values for NPC diagnosis	Wang et al. (2019)
	miR-18a-5p	Up	Has significant clinical values for NPC diagnosis	Wang et al. (2019)
LCC	miR-24	–	Suppresses mitochondrial mt-Nd2, Snora75	Michael et al. (2017)

### Diagnosis, prognosis, and treatment of TEP RNA and NSCLC

**Fig. 3:**
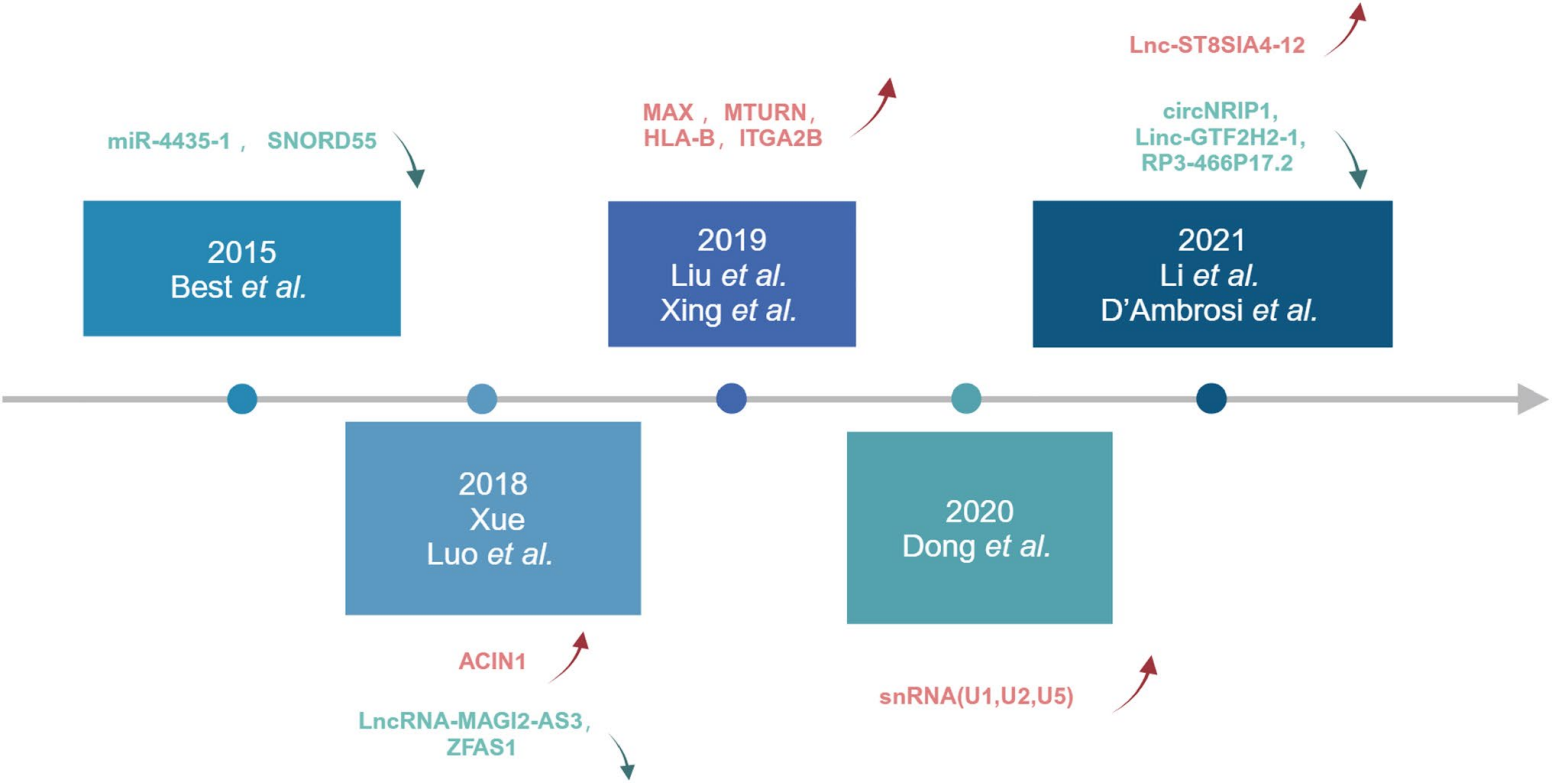
The research progress of TEP RNA in NSCLC in the past decade. Since 2015, people have gradually begun to study the diagnostic applications of TEPs in NSCLC. Best et al. found in 2015 that miR-4435-1 and SNORD55 in TEPs were low expressed in NSCLC. Xue and Luo et al. found in 2018 that ACIN1 in TEPs was highly expressed in NSCLC, while LncRNA-MAGI2-AS3 and ZFAS1 were low expressed in NSCLC. In 2019, Liu and Xing et al. found that MAX, MTURN, HLA-B, and ITGA2B in TEPs were highly expressed in NSCLC. In the most recent year in 2021, Li and D’Ambrosi et al. found that Lnc-ST8SIA4-12 in TEPs was highly expressed, while circNRIP1, Linc GTF2H2-1, RP3-466P17.2 were low expressed in NSCLC. Created with BioRender.com

The applications of TEP RNA in the diagnosis of NSCLC have been investigated since 2015 (Fig. 3). Zhang et al. performed RNA-seq on platelets from NSCLC patients and conducted differential gene expression analysis and subgroup analysis (Zhang et al. 2018). The results indicated that platelet RNA-seq can be used not only for distinguishing NSCLC from individuals, but also for the classification of lung cancer. In addition, Best et al. used a particle swarm optimization (PSO) enhanced algorithm to screen and verify TEP RNA for NSCLC patient staging (Best et al. 2017). Then Sheng et al. performed the IFS method to screen 48 biomarkers from TEP RNA-seq data of NSCLC patients and conducted cross validation. They found that liquid biopsy of 48-genes contributes to the early diagnosis and prolonged survival of NSCLC (Sheng et al. 2018). On the basis of previous sequencing, researchers conducted further research and validation on TEP RNA related genes and discovered their clinical applications. In NSCLC, multiple TEP RNAs are upregulated or downregulated, serving as both oncogenes and tumor suppressors. Table 2 lists important TEP RNAs related to the development of NSCLC.

**Table 2:** Roles of TEP RNA in the occurrence and progression of NSCLC

TEP RNA	Dysregulation type	Function	References
MAX	Up	Coordinates with NF-κB to promote Fas ligand expression	Liu et al. (2019b)
MTURN	Up	Participates in MAPK/ERK, SAPK/ JNK and NF-κB signaling pathway	Liu et al. (2019b)
HLA-B	Up	Presents endogenous antigens to CD8-positive T cells	Liu et al. (2019b)
ACIN1	Up	Induces cell death through the interaction with AAC-11	Xue et al. (2018)
ITGA2B	Up	Participates in PI3K signaling and causes gene mutations	Xing et al. (2019)
snRNA(U1,U2,U5)	Up	–	Dong et al. (2020)
Lnc-ST8SIA4-12	Up	–	Li et al. (2021)
LncRNA-MAGI2-AS3	Down	Upregulates cytokine signaling 1 by sponging miR-155	Luo et al. (2018)
ZFAS1	Down	LncRNA ZFAS1/miR-590-3p axis is involved in NSCLC cell proliferation	Luo et al. (2018)
circNRIP1	Down	–	D’Ambrosi et al. (2021b)
Linc-GTF2H2-1	Down	–	Li et al. (2021)
RP3-466P17.2	Down	–	Li et al. (2021)
miR-4435–1	Down	–	Best et al. (2015)
SNORD55	Down	–	Best et al. (2015)

Research has found that TEP mRNA of MAX, MTURN, and HLA-B groups are highly expressed in NSCLC. All three groups of TEP RNA can be used to evaluate the effect of chemotherapy (Liu et al. 2019b). MAX helps members of the transcription factor network bind to DNA, and tissues lacking MAX-interacting protein MNT are prone to tumor formation (Hurlin and Huang 2006). Although MAX acts as a tumor suppressor in small cell lung cancer (Augert et al. 2020), it can also interact with NF-κB synergistically to promote Fas ligand expression in NSCLC and promote Fas related immune escape mechanism (Bennett et al. 1998; Kasibhatla et al. 2000; Wiener et al. 2004). Furthermore, MYC can serve as a therapeutic target for MAX mutated pheochromocytoma, and we can continue to study the application of MYC in NSCLC (Cascón and Robledo 2012; Massó-Vallés et al. 2020). MTURN is involved in MAPK/ERK, SAPK/JNK and NF-κB signaling pathways and is highly expressed in platelets of NSCLC patients (Sun et al. 2014). Consistent with this, the diagnostic efficiency of TEP MTURN in female patients is extremely high (Liu et al. 2019b). Lian et al. constructed a prognostic TF-methylation-gene network by single-factor Cox regression analysis, analyzed the methylation sites and genes of lung adenocarcinoma patients, and concluded that MTURN was downregulated in LUAD samples. However, the expression and regulatory mechanism of MTURN in NSCLC is still unclear and need to be further studied (Lian et al. 2022). It is reported that HLA is highly expressed in NCSLC and plays an important role in p300 mediated p53 acetylation (Goodman and Smolik 2000; Sasaki et al. 2007, p. 5; Tsukahara et al. 2009). In early stage of lung adenocarcinoma, ANIN1 interaction with AAC-11 and promotes tumor cell development and malignant transformation under high DNA methylation level (Shu et al. 2006; Rigou et al. 2009). We have learned from recent research that ITGA2B mutations can lead to changes in the PI3K signaling pathway, promote the occurrence of cancer and predict poor prognosis in large cell lung cancer (Guo et al. 2023). Besides, the high expression of ITGA2B in platelets of NSCLC patients has been confirmed by many researchers (Xing et al. 2019; Negahdari et al. 2020; Lecot et al. 2022). Dong et al. found that snRNA (U1, U2, U5) were low expressed in platelets of lung cancer patients, and this study is the only one that has applied TEP snRNA to the diagnosis of NSCLC (Dong et al. 2020). Although the mechanism is not completely clear, some scholars have pointed out that RNU2 is highly expressed in lung cancer patients and can be used as a bi-functional ncRNA to process a 19-22nt fragment for the detection of NSCLC (Mazières et al. 2013; Köhler et al. 2016). The discovery of anti-U1-A snRNP antibody in lung cancer patients has further promoted the application of RNU1 in the diagnosis of NSCLC (Zhang et al. 2005). lncRNA-MAGI2-AS3, as an oncogene, is low expressed in platelets of NSCLC patients (Luo et al. 2018). Researchers and scholars have studied the mechanism: lncRNA MAGI2-AS3 inhibits the proliferation, migration and invasion of NSCLC cells by regulating miR-374a/b-5p/CADM2, miRNA-23a-3p/PTEN and miR-629-5p/TXNIP axis and up-regulating RECK and cytokine signaling 1 in combination with miR-25 and miR-15583 (Hao and Yang 2019; He et al. 2020; Li et al. 2020). However, the lncRNA ZFAS1, which regulates NSCLC cell proliferation through ZFAS1/miR-590-3p axis, was found to be downregulated in TEPs of NSCLC patients (Luo et al. 2018; Zhou et al. 2020). This may be influenced by factors from different gene sources, and the same gene may exhibit different expression trends in pathological tissues and TEPs.

Although researchers have found high expression of NRC-ST8SIA4-12, and low expression of circNRIP1, linc-GTF2H2-1, RP3-466P17.2, miR-4435-1, and SNORD55 in NSCLC TEPs, the mechanism of action of these genes in NSCLC remains unclear (Table 2). We need to further investigate the mechanisms in order to better apply the biomarkers to the diagnosis and treatment of NSCLC.

## Methods for detecting platelet RNA

**Fig. 4:**
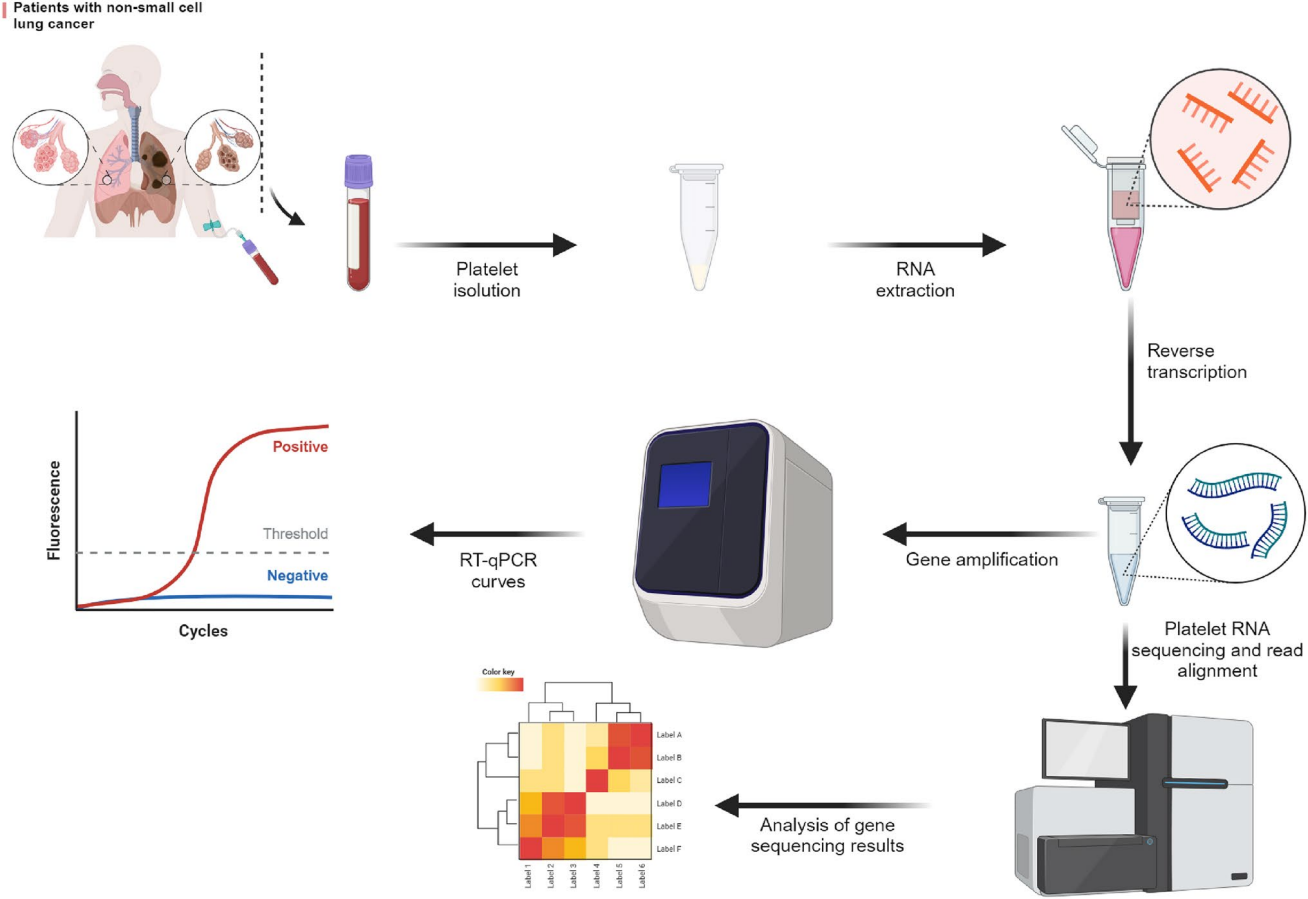
Steps for TEP RNA study. Collect whole blood from the patient and store it with EDTA-K2 before separating platelets through two-step of low-speed centrifugation. Extract platelets RNA and perform NGS second-generation sequencing or real-time fluorescence quantitative PCR. Analyze the relationship between platelet RNA and NSCLC based on sequencing results or gene expression levels. Created with BioRender.com

Based on existing studies and databases, we can further conduct confirmatory experiments on differentially expressed genes in NSCLC patients (Fig. 4). After collecting EDTA-K2 anticoagulants from a large number of NSCLC patients, platelets were isolated from plasma. The main extraction methods of platelets include washing, gradient density centrifugation, gel filtration, immunomagnetic bead separation, flow cytometry (FCM), etc. (Amisten 2012). Even if white blood cells can be removed by methods such as CD45 or magnetic bead sorting, they are still cumbersome, costly and may reduce platelet production. Therefore, gradient centrifugation has been included in thromboSeq protocol and can be used extensively in clinical trials (Rolf et al. 2005; Coppinger et al. 2007; Bray et al. 2013). To ensure platelet purity, an optical microscope was used to count platelet suspensions (Best et al. 2015) (There are less than 5 white blood cells per 1 million platelets). Platelet RNA was extracted and reverse-transcribed into cDNA for quantitative detection by RT-qPCR or NGS. Then, the differentially expressed genes in platelets will be analyzed based on Ct values or sequencing results.

## Conclusions and future perspectives

There exists an intricate interplay between tumor cells and platelets. On the one hand, tumors and the surrounding environment exert an influence on the quantity, size, molecular omics, and function of platelets. On the other hand, platelets affect occurrence, development, and metastasis of tumor. Platelets and PMPS promote the development and progression of tumors by creating an inflammatory environment (Michael et al. 2017), stimulating angiogenesis, facilitating immune evasion, and modulating signaling pathways (Plantureux et al. 2020). Platelets also possess the capability to prevent tumor cell proliferation, indicating the necessity for comprehensive analysis of platelet function based on tumor type, disease stage, local microenvironment, and other pertinent factors. Currently, a large number of studies have confirmed significant differences in the expression of TEP RNA in platelets between NSCLC patients and healthy individuals. It is imperative to continuously explore the diagnostic value of TEP RNA in NSCLC.

Based on the bidirectional interaction between tumor cells and platelets, platelets are considered as emerging targets with significant potential in tumor diagnosis and treatment. The integration of NSCLC platelet-related markers with other NSCLC markers can enhance existing tumor diagnosis and prognostic evaluation systems, providing broad prospects in tumor screening, disease monitoring, and prognosis assessment. Beyond that, targeted inhibition of platelet function represents a feasible anti-tumor treatment strategy. In the development of anti-tumor nanomedicine, platelets exhibit excellent biocompatibility, tumor targeting, safety, and drug loading ability. The use of platelet simulation targeting agents can synergistically eradicate NSCLC cells through various methods, such as chemotherapy, phototherapy, radiotherapy, and calcium overload. Platelet-related tumor markers and targeted anti-tumor drugs have great potential in the field of tumor diagnosis and treatment, providing a promising pathway for achieving breakthroughs in precision oncology.

However, the application of platelets in the diagnosis and treatment of NSCLC is still in its early stages. In addition to the drawbacks of low platelet count and complex experimental processes, the diagnostic accuracy of TEP RNA-seq for cancer in different populations still needs to be improved and validated. For example, platelet RNA sequencing has poor diagnostic accuracy for detecting occult cancers in patients with unprovoked VTE (Best et al. 2015). We need to conduct a comprehensive analysis of TEP RNA from various aspects, including age, gender, and factors that contribute to NSCLC patients. It is important to analyze different patient situations separately, rather than just focusing on tumor phenotype. More importantly, in the clinical transformation process, we must pay attention to the risk of adverse reactions such as bleeding and off target of platelet-related anti-tumor drugs. To maximize the benefit of cancer patients, the molecular mechanism of platelet-tumor interaction needs to be further explored. Platelet-related tumor biomarkers and anti-tumor targeted drugs have broad prospects in tumor diagnosis and treatment, and are expected to bring significant breakthroughs for precise tumor treatment. Therefore, our future research direction will not be limited to the diagnostic value of TEP RNA in NSCLC, but should also extended to the intervention effect of TEP RNA.

## Data Availability

Not applicable.

## Abbreviations

NSCLC: Non-small cell lung cancer
LDCT: Low-dose computed tomography
LB: Liquid biopsy
CTCs: Circulating tumor cells
EVs: Extracellular vesicle
MKs: Megakaryocytes
RBP: RNA-binding protein
TEPs: Tumor-educated platelets
HBC: Hepatobiliary cancer
CRC: Colorectal cancer
GBM: Glioblastoma
PAAD: Pancreatic cancer
BC: Breast cancer
OC: Ovarian cancer
ESCA: Esophageal cancer
MPN: Myeloproliferative neoplasms
NPC: Nasopharyngeal carcinoma
LCC: Lung and colon carcinoma.
PLR: Platelet-to-lymphocyte ratio
OS: Overall survival
MPV: Mean platelet volume
PFS: Progression-free survival
irAEs: Immune-related adverse events
ICIs: Immune checkpoint inhibitors
VEGF: Vascular endothelial growth factor
PDGF: Platelet-derived growth factor
MAX: MYC-associated protein X
MTURN: Maturin, neural progenitor differentiation regulator homolog
HLA-B: Human leukocyte antigen B
ACIN1: Apoptotic chromatin condensation inducer 1
ITGA2B: Integrin, alpha 2b (platelet glycoprotein IIb of IIb/IIIa complex, antigen CD41)
ZFAS1: ZNFX1 antisense RNA 1
SNORD55: Small nucleolar RNA C/D box 55
